# Supplementary material for: What does it take to learn the rules of RNA base pairing? A lot less than you may think
Source: bioRxiv. 2025 Aug 2:2025.07.31.668042. Preprint. [Version 1] doi: 10.1101/2025.07.31.668042 (PMC12324431; doi:10.1101/2025.07.31.668042)
Supplement: Supplement 1 [file media-1.gz › supplemental_material/src/rscape_v2.6.0.tar.gz!/rscape_v2.6.0/lib/hmmer/documentation/userguide/inclusions/daemon-results.pdf]

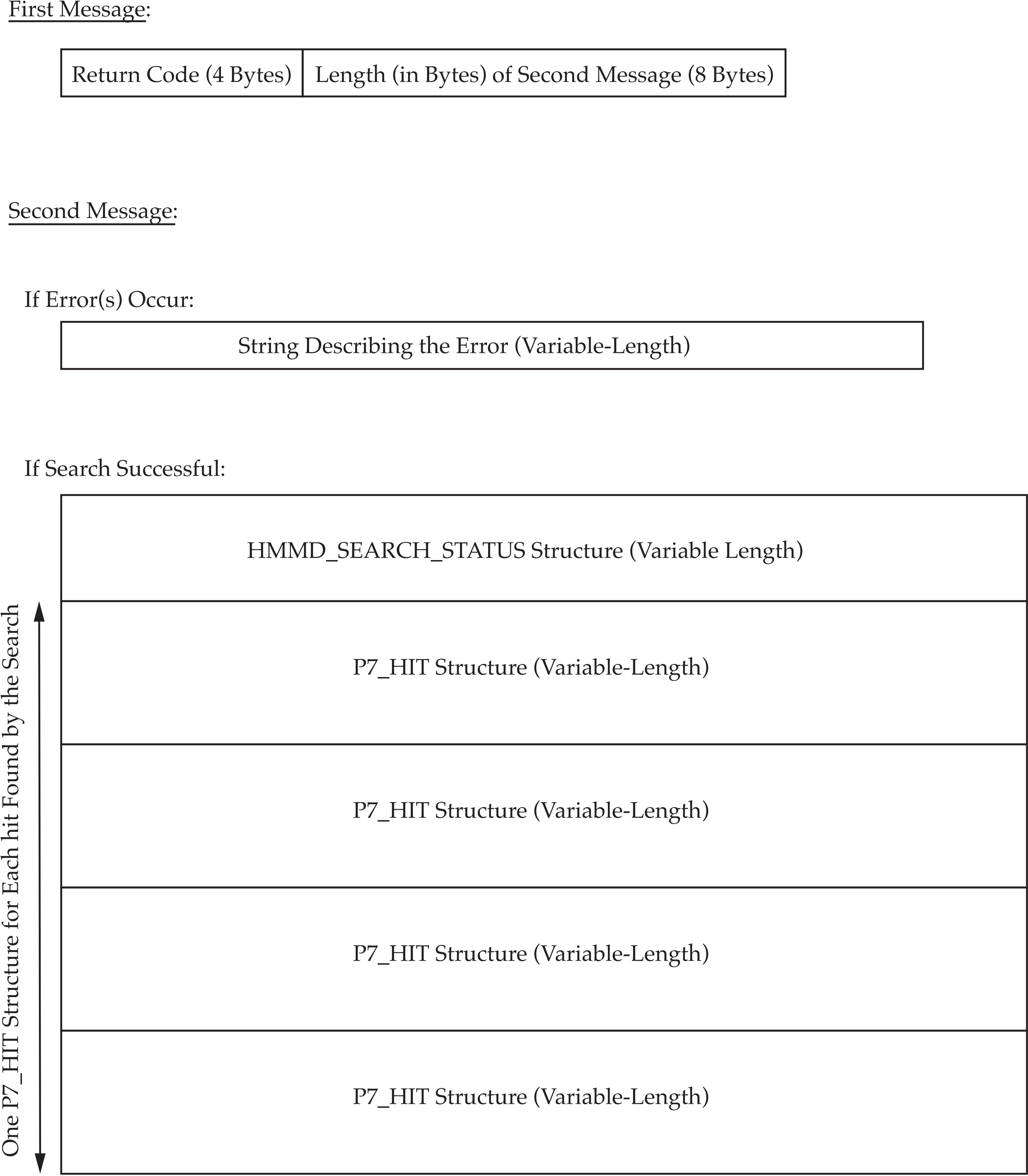
First Message:
Return Code (4 Bytes)
Length (in Bytes) of Second Message (8 Bytes)
Second Message:
If Error(s) Occur:
String Describing the Error (Variable-Length)
If Search Successful:
HMMD_SEARCH_STATUS Structure (Variable Length)
P7_HIT Structure (Variable-Length)
P7_HIT Structure (Variable-Length)
P7_HIT Structure (Variable-Length)
P7_HIT Structure (Variable-Length)
One P7_HIT Structure for Each hit Found by the Search
